# Cell-free DNA epigenomic profiling enables noninvasive detection and monitoring of translocation renal cell carcinoma

**DOI:** 10.1172/JCI195725

**Published:** 2026-02-02

**Authors:** Simon Garinet, Karl Semaan, Jiao Li, Ze Zhang, Prathyusha Konda, Ananthan Sadagopan, John Canniff, Noa Phillips, Kelly Klega, Medha Pandey, Hunter Savignano, Matthew P. Davidsohn, Kevin Lyons, Alessandro Medda, Prateek Khanna, Mingkee Achom, Brad J. Fortunato, Rashad Nawfal, Razane El Hajj Chehade, Jillian O’Toole, Jack Horst, Dory Freeman, Rachel Trowbridge, Cindy H. Chau, William D. Figg, Jacob E. Berchuck, Brian D. Crompton, Ji-Heui Seo, Toni K. Choueiri, Matthew L. Freedman, Sylvan C. Baca, Srinivas R. Viswanathan

**Affiliations:** 1 Department of Medical Oncology, and; 2 Center for Functional Cancer Epigenetics, Dana-Farber Cancer Institute, Boston, Massachusetts, USA.; 3 Department of Medicine, Brigham and Women’s Hospital, Boston, Massachusetts, USA.; 4 Dana-Farber/Boston Children’s Cancer and Blood Disorders Center, Harvard Medical School, Boston, Massachusetts, USA.; 5 Department of Medicine, Harvard Medical School, Boston, Massachusetts, USA.; 6 Genitourinary Malignancies Branch, Center for Cancer Research, National Cancer Institute, National Institutes of Health, Bethesda, Maryland, USA.; 7 Winship Cancer Institute, Emory University, Atlanta, Georgia, USA.; 8 Cancer Program, Broad Institute of MIT and Harvard, Cambridge, Massachusetts, USA

**Keywords:** Clinical Research, Oncology, Biomarkers, Cancer, Molecular diagnosis

## Abstract

*TFE3* translocation renal cell carcinoma (tRCC), an aggressive kidney cancer driven by *TFE3* gene fusions, is frequently misdiagnosed owing to morphologic overlap with other kidney cancer subtypes. Conventional liquid biopsy assays that detect tumor DNA via somatic mutations or copy number alterations are unsuitable for tRCC since it often lacks recurrent genetic alterations and because fusion breakpoints are highly variable between patients. We reasoned that epigenomic profiling could more effectively detect tRCC because the driver fusion constitutes an oncogenic transcription factor that alters gene regulation. By defining a TFE3-driven epigenomic signature in tRCC cell lines and detecting it in patient plasma using ChIP-seq, we distinguished tRCC from clear-cell RCC (AUC = 0.86) and samples of individuals without evidence of cancer (AUC = 0.92) at low tumor fractions (<1%). This work establishes a framework for noninvasive epigenomic detection, diagnosis, and monitoring of tRCC, with implications for other mutationally quiet, fusion-driven cancers.

## Abstract 2

Plasma epigenomic profiling detects tRCC in plasma with high accuracy, enabling non-invasive diagnosis and monitoring of this fusion-driven kidney cancer.

## Graphical abstract

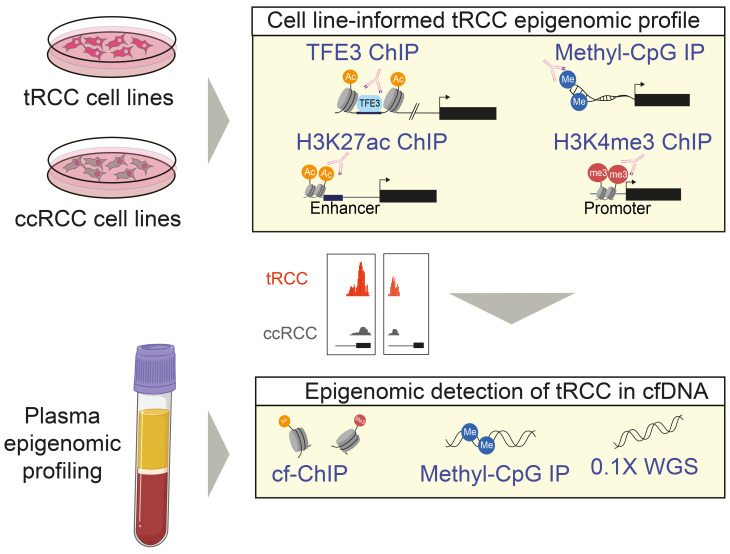

## Introduction

Translocation renal cell carcinoma (tRCC) is an aggressive subtype of RCC driven by a gene fusion involving an MiT/TFE family transcription factor, most commonly *TFE3*. tRCC comprises up to 5% of RCCs in adults (1, 2) and over 50% of RCCs in children (3, 4). Despite harboring a distinctive genetic landscape, tRCC shares histologic overlap with other RCC subtypes (5), complicating its accurate diagnosis and an estimation of its true incidence. While *TFE3* gene fusions can be detected by break-apart FISH (6) or next-generation sequencing assays, these tests are not always routinely performed in clinical practice (7–9). Moreover, the variety of *TFE3* fusion partners and genomic breakpoint locations leads to an appreciable false negative rate with these genetic detection methods (10–13). This, along with the inherent heterogeneity among kidney cancers, which number over 40 different subtypes, creates a critical need for methods that allow for accurate diagnosis and monitoring of tRCC.

Circulating tumor DNA (ctDNA) assays have revolutionized the ability to noninvasively survey molecular features of a patient’s tumor (14–16). To date, most ctDNA assays have focused on the detection of genomic alterations, such as point mutations or copy number variants (17). However, such assays are not suitable for the detection or monitoring of tRCC, which often harbors no genetic alterations apart from the driver fusion (9, 18). Moreover, fusion breakpoints are challenging to identify in ctDNA (19), and this is particularly the case in tRCC, where breakpoint locations and *TFE3* partner genes vary widely between patients (9, 18, 20, 21). Indeed, approved cell-free DNA–based (cfDNA-based) liquid biopsy tests often do not include *TFE3* fusions in the gene panel.

Epigenomic profiling of circulating nucleosomes in plasma has recently emerged as a powerful tool for cancer detection and subtyping that provides orthogonal information to mutation-based methods (22–26). Using an immunoprecipitation-based approach — cell-free ChIP-seq (cf-ChIP) — histone modifications and DNA methylation can be profiled from circulating nucleosomes that originate from cancer cells, enabling the noninvasive measurement of tumor gene regulatory programs in plasma (22, 24, 27–29).

Given that tRCC is driven by a gene fusion that results in the expression of an oncogenic transcription factor, we hypothesized that cf-ChIP would be uniquely well suited to detect tRCC-specific profiles of regulatory element activity (30, 31), an approach that could also be applied to other fusion-driven malignancies.

## Results

**Figure 1:**
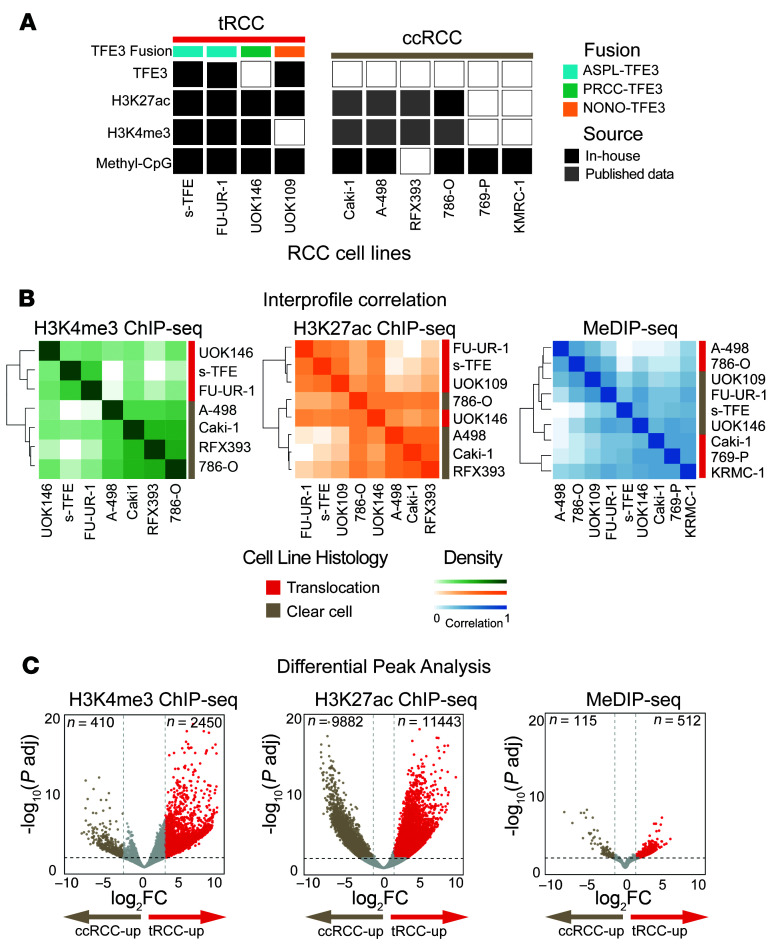
Cell line–informed epigenomic signature of tRCC. (**A**) Epigenomic datasets generated from 4 tRCC (s-TFE, FU-UR-1, UOK109, and UOK146) and 6 ccRCC (Caki-1, A-498, RFX393, 786-O, 769-P, and KMRC-1) cell lines, either in-house or in a previously published study (69, 81). (**B**) Unsupervised hierarchical clustering of the H3K4me3 ChIP-seq, H3K27ac ChIP-seq, and MeDIP-seq consensus peaks across tRCC and ccRCC cell lines analyzed in this study. (**C**) Volcano plots showing differentially marked peaks between tRCC and ccRCC cell lines for H3K4me3 ChIP-Seq, H3K27ac ChIP-seq, and MeDIP-seq. Thresholds for significance were set at FDR-q < 0.01 and log_2_FC > 1 for H3K27ac and MeDIP and > 2 for H3K4me3.

To define an epigenomic signature associated with the TFE3 fusion, we first profiled or reanalyzed 27 epigenomic libraries from 4 tRCC and 6 clear-cell RCC (ccRCC) lines (Figure 1A; see Methods). We performed ChIP-seq for 2 posttranslational histone modifications (H3K4me3 and H3K27ac) as well as methylated CpG dinucleotide immunoprecipitation and sequencing (MeDIP-seq). H3K4me3 is enriched at active gene promoters (32), and H3K27ac is enriched at active gene promoters and enhancers (33), while DNA methylation is associated with promoter silencing (34). Across all RCC cell lines, a median of 29,588 peaks (range 25,314–30,076) were captured by H3K4me3 ChIP-seq, a median of 57,226 peaks (range 44,470–73,400) by H3K27ac ChIP-seq, and a median of 229,624 peaks (range 111,904–297,344) by MeDIP-seq (Supplemental Figure 1; supplemental material available online with this article; https://doi.org/10.1172/JCI195725DS1).

Unsupervised hierarchical clustering and principal component analyses of consensus H3K4me3 and H3K27ac peaks revealed clear segregation of tRCC and ccRCC cell lines, while DNA methylation profiles were minimally discriminatory between tRCC and ccRCC (Figure 1B and Supplemental Figure 1; see Methods), even when restricted to CpG islands (Supplemental Figure 1). This may be in part because methylation patterns tend to be more stable and often highlight lineage identity, which may be similar between ccRCC and tRCC (13, 31, 35–37), whereas histone marks, particularly active modifications, can more dynamically reflect transcriptional changes driven by the TFE3 fusion (9, 33).

**Figure 2:**
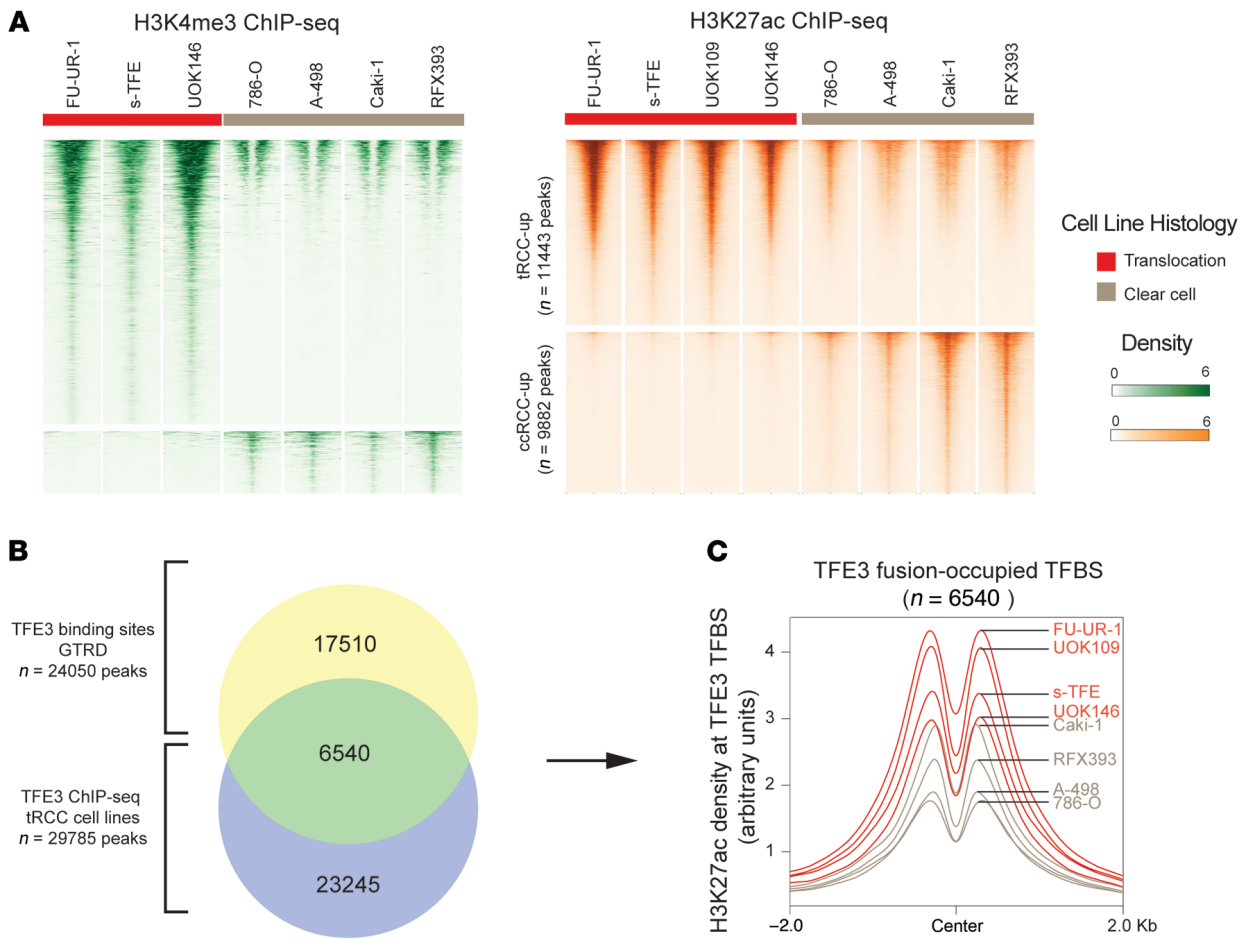
Definition of TFE3 fusion–occupied sites in tRCC. (**A**) Heatmaps of normalized H3K27ac and H3K4me3 tag densities at differentially marked regions between tRCC and ccRCC cell lines shown over a window ±2 kb from peak center. (**B**) Schema for identifying 6,540 TFE3 fusion–occupied TFBSs through the intersection of known TFE3 TFBSs (GTRD) and TFE3 fusion peaks via TFE3 ChIP-seq in tRCC cell lines. (**C**) Aggregated H3K27ac density at 6,540 TFE3 fusion–occupied TFBSs across RCC cell lines profiled in this study, showing increased signal in tRCC cell lines (red) compared with ccRCC cell lines (gray).

We next sought to identify regulatory elements with differential epigenetic modifications in tRCC versus ccRCC cells. Overlapping peaks across samples were merged for each mark, creating a consensus set of 26,529, 63,322, and 342,285 peaks for the H3K4me3, H3K27ac, and MeDIP profiles, respectively. Via differential peak analysis of ChIP-seq data using the DiffBind R package (38), we identified 2,860 differential H3K4me3 peaks (of which 2,450 were enriched in tRCC [tRCC-up]; FDR *q* value [FDR-q] < 0.01 and log_2_fold-change [log_2_FC] > 2) and 21,325 differential H3K27ac peaks (of which 11,443 were tRCC-up; FDR-q < 0.01 and log_2_FC > 1) (Figure 1C and Figure 2A; see Methods). By contrast, among MeDIP-seq peaks, we identified only 627 differentially methylated regions (DMRs) between tRCC and ccRCC (FDR-q < 0.01 and log_2_FC > 1; Figure 1C and Supplemental Figure 1; see Methods), consistent with the weak segregation observed in unsupervised hierarchical clustering. Motif analysis of the 11,443 H3K27ac tRCC-up peaks identified significant enrichment for sequences bound by TFE3 and its paralog, MITF, which share consensus binding sites (39). This suggests that H3K27ac tRCC-up sites include regulatory elements activated by direct binding of TFE3 fusions (Supplemental Figure 2) and is consistent with recent reports that TFE3 fusions may facilitate the organization of enhancer loops and transcriptional activation (31, 40–43).

Next, we sought to refine our epigenetic signature by incorporating transcriptionally active sites directly bound by the TFE3 fusion in tRCC. First, to obtain a robust consensus set of TFE3 fusion binding sites, we intersected 24,050 WT TFE3 transcription factor binding sites (TFBSs) derived from 2 non-RCC cell lines (LoVo, a colorectal cancer cell line, and HepG2, a hepatocellular carcinoma cell line) in the Gene Transcription Regulation Database (GTRD) with 29,785 TFE3 TFBSs identified via ChIP-seq in 3 tRCC cell lines representing 2 distinct TFE3 fusions (44). This resulted in a final set of 6,540 sites that we deemed fusion-occupied TFBSs (Figure 2B; see Methods). Assessing aggregated H3K27ac signal across these 6,540 fusion-occupied TFBSs revealed a higher signal in all tRCC cell lines compared with ccRCC cell lines (Figure 2, A and C). Importantly, this difference in signal intensity was less pronounced when considering all TFBSs from GTRD (*n* = 24,050) or the fusion nonoccupied binding sites, which did not overlap with TFE3 fusion ChIP-seq peaks (*n* = 17,510) (Supplemental Figure 2), underscoring the importance of building a robust consensus set of TFE3 fusion–occupied TFBSs. Furthermore, only 853 of the 6,540 fusion-occupied TFBSs overlapped with the H3K27ac tRCC-up sites (*n* = 11,443) identified using DiffBind (Supplemental Figure 2), suggesting that these 2 methods identify partly nonoverlapping regulatory sites associated with tRCC. We sought to evaluate the specificity of our 3 sets of sites for tRCC compared with other RCC subtypes. At the fusion-occupied TFBSs and H3K27ac and H3K4me3 tRCC-up sites, we computed the aggregated H3K27ac and H3K4me3 ChIP-seq signal in tRCC, ccRCC, and papillary RCC (pRCC) cell lines (Supplemental Figure 3). The signal for pRCC cell lines was similar to that observed in ccRCC and trended lower than that in tRCC. We extended this analysis using publicly available H3K27ac data from 6 pRCC, 12 chRCC, and 12 ccRCC tumor samples (45). Here again, the signals in pRCC and chRCC were comparable with those observed in ccRCC previously.

**Figure 3:**
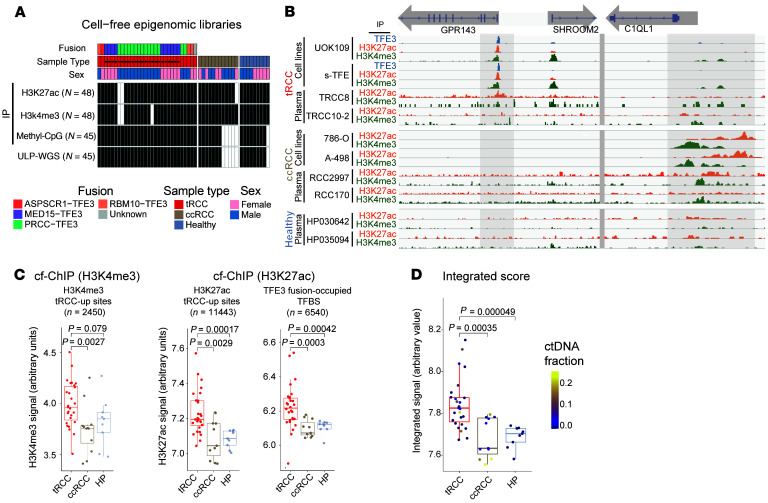
Detection of a tRCC-specific epigenomic signature via plasma cf-ChIP. (**A**) Epigenomic datasets generated from 51 plasma samples collected from patients with metastatic tRCC (*N* = 30; 10 patients), metastatic ccRCC (*N* = 12; 12 patients), or those acting as healthy controls (*N* = 9; 9 individuals). (**B**) Integrative Genomics Viewer tracks from ChIP-seq profiles for H3K4me3, H3K27ac, and TFE3 in cell lines (tRCC: UOK109, s-TFE; ccRCC: 786-O, A-498) and plasma samples at representative tRCC-selective (*GPR143*) or ccRCC-selective (*C1Q1L*) loci. (**C**) Aggregated cf-ChIP signal compared among tRCC, ccRCC, and healthy plasma samples for the following marks; the box plots quantify the AUCs for each histone mark (left to right): H3K4me3 signal at cell line–informed H3K4me3 tRCC-up sites was significantly higher in 28 tRCC samples from 10 patients compared with 11 ccRCC samples from 11 patients (*P* = 0.0027) and showed a trend when compared with 9 healthy control samples (*P* = 0.079); H3K27ac signal at cell line–informed H3K27ac tRCC-up sites was significantly higher in 27 tRCC samples from 10 patients compared with 12 ccRCC samples from 12 patients (*P* = 0.0029) and with 9 healthy control samples from 9 individuals (*P* = 0.00017); H3K27ac signal at TFE3 fusion–occupied TFBSs was significantly higher in 27 tRCC samples from 10 patients compared with 12 ccRCC samples from 12 patients (*P* = 0.0003) and with 9 healthy control samples from 9 individuals (*P* = 0.00042). *P* values were determined by Wilcoxon’s test. (**D**) Comparison of TIESs of cf-ChIP H3K4me3 and H3K27ac signals at cell line–informed sites (H3K4me3 tRCC-up peaks, H3K27ac tRCC-up peaks, and TFE3 fusion–occupied binding sites) among tRCC, ccRCC, and healthy plasma, with samples color-scaled according to tumor fraction. TIES was significantly higher in 27 tRCC samples from 10 patients compared with 11 ccRCC samples from 11 patients (*P* = 0.00035) and with 9 healthy control samples from 9 individuals (*P* = 0.000049). HP, healthy plasma. *P* values were determined by Wilcoxon’s test. The box plots depict the minimum and maximum values (whiskers), the upper and lower quartiles, and the median.

Having identified a tRCC-specific epigenomic signature in cell line models, we next evaluated its ability to discriminate plasma from patients with tRCC, ccRCC, and those serving as healthy controls using cf-ChIP. We profiled 141 epigenomic libraries from 51 plasma samples from patients with tRCC (*N* = 30 samples), ccRCC (*N* = 12), and healthy individuals (*N* = 9) (Figure 3A). cf-ChIP revealed increased signals at tRCC-up or ccRCC-up peaks in RCC plasma that were absent in plasma from healthy volunteers (Figure 3B). For example, in tRCC plasma, H3K4me3 and H3K27ac signals were elevated at the *GPR143* gene locus, a tRCC-specific peak and TFE3-fusion target gene (46). Conversely, we observed an increased H3K4me3 and H3K27ac signal at the *C1QL1* gene locus in ccRCC plasma samples. These findings were concordant with the published RNA-seq data in ccRCC and tRCC cell lines, as well as in tRCC and ccRCC tumor samples from a cohort of patients with metastatic ccRCC (47) (Supplemental Figure 4).

Given that *GPR143* and *C1QL1* appeared to be highly selective marker genes for tRCC and ccRCC, respectively, we also attempted to measure their expression directly in the blood by profiling circulating tumor cells (CTCs). A cohort of 10 patients with metastatic ccRCC, 7 with metastatic tRCC, and 1 serving as a healthy control was sampled for CTCs using isolation on the TellDx CTC system (see Methods). We extracted RNA from pelleted samples and aimed to detect tRCC-specific (*GPR143* and *TRIM63*) or ccRCC-specific (*C1QL1*) transcripts by digital droplet PCR (ddPCR) after whole transcriptome amplification. While these transcripts could be detected in healthy blood spiked with RNA derived from tRCC or ccRCC cell lines, no signal was detectable in the tRCC or ccRCC patient samples (Supplemental Figure 4). Of note, 4 patients were sampled for both cfDNA and CTC isolation, with 2 of them being sampled from the same blood draw. These results suggest that cf-ChIP can infer tRCC gene expression programs even when they are not detectable by standard methods for profiling CTCs.

Next, to develop an epigenome-wide cf-ChIP signature for detecting tRCC, we compared H3K4me3 and H3K27ac coverage in patient plasma at the 2,450 H3K4me3 tRCC-up and 11,443 H3K27ac tRCC-up peaks, informed by cell line profiling. Aggregated coverage at both H3K4me3 and H3K27ac tRCC-up sites was increased in plasma from patients with tRCC compared with patients with ccRCC (*P* = 0.0027 and 0.0029, respectively) or those serving as healthy controls (*P* = 0.079 and 0.00017, respectively) (Figure 3C). Conversely, the aggregated methylation signal in plasma measured using cf-MeDIP at tRCC DMRs derived from cell lines or The Cancer Genome Atlas (TCGA) methylation data did not distinguish tRCC and ccRCC plasma samples (Supplemental Figure 5). Finally, H3K27ac coverage at the 6,540 TFE3 fusion–occupied binding sites showed higher discriminating power versus ccRCC (*P* = 0.0003) and versus healthy patients (*P* = 0.00042), perhaps due to WT TFE3 activity in white blood cells, particularly macrophages, where MiT/TFE genes can be active (48) (Figure 3C).

**Figure 4:**
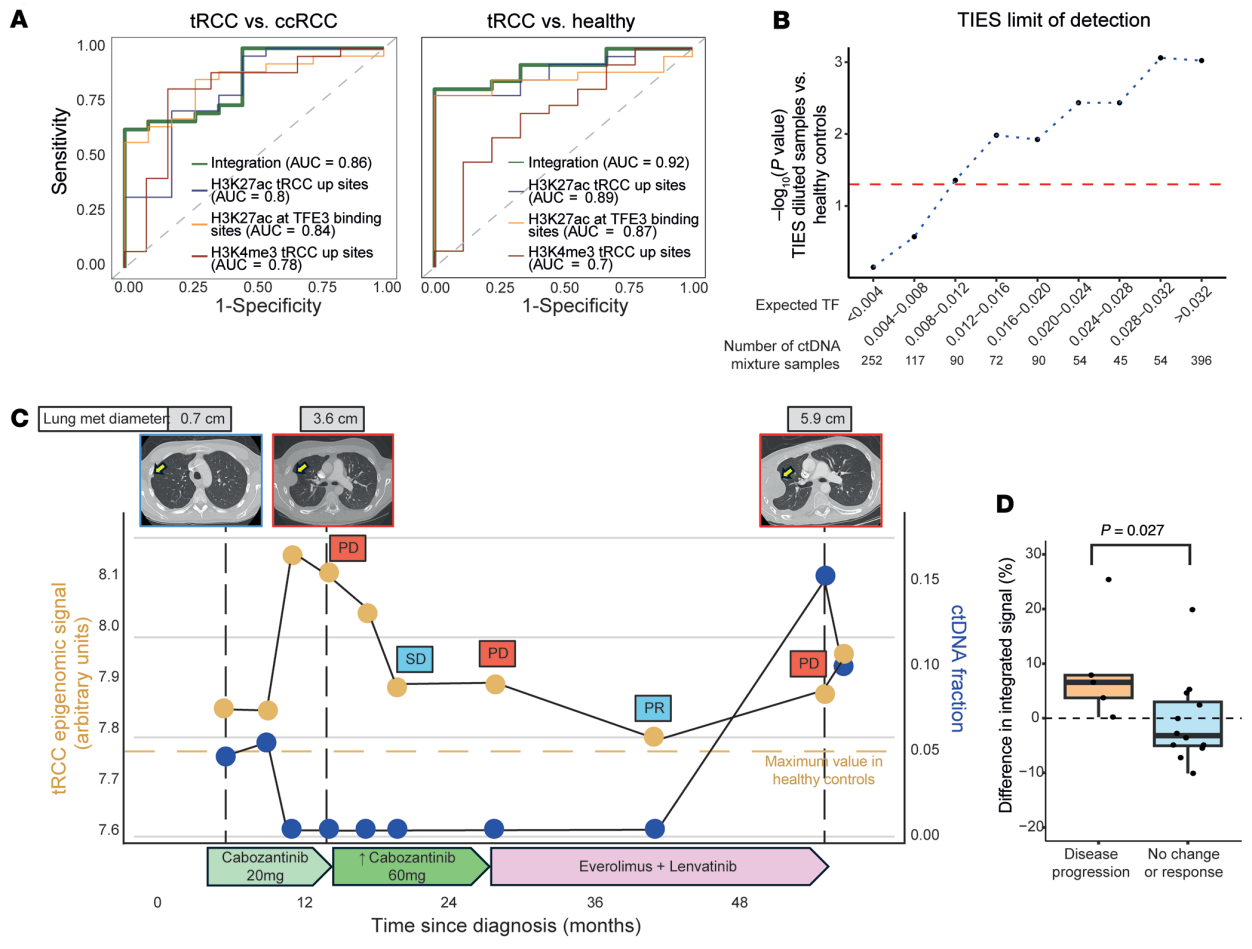
Detection and monitoring of tRCC using cf-ChIP. (**A**) Classifier assessing individual cf-ChIP H3K4me3 and H3K27ac signals at cell line–informed sites – H3K4me3 tRCC-up peaks, H3K27ac tRCC-up peaks, and TFE3 fusion–occupied binding sites — and evaluating their combined performance in distinguishing tRCC from ccRCC (left) and tRCC versus healthy plasma (right). (**B**) Estimation of TIES limit of detection. Ten tRCC samples (all with tumor fraction > 3% by ichorCNA) were diluted in silico by adding reads from 9 healthy plasma samples at 0.9, 0.8, 0.7, 0.6, 0.5, 0.4, 0.3, 0.2, 0.1, 0.05, and 0.01 ratios. An estimated tumor fraction (TF) was assigned for each dilution, equal to the TF calculated by ichorCNA for the tRCC sample multiplied by the portion of tRCC reads for that dilution. For a given bin of estimated TF, TIESs for all tRCC samples were pooled and compared with the healthy plasma value (Wilcoxon’s rank sum test). Red dashed line indicates the threshold of significance (*P* = 0.05). (**C**) Longitudinal tracking of the TIES (orange) and ctDNA fraction (blue) in a patient with tRCC. Radiographic changes in an index lesion (pleural metastasis) are provided, as are timings and doses of administered systemic therapies. SD, stable disease; PR, partial response; PD, progressive disease. (**D**) Percent change in TIES between consecutive plasma draws was significantly higher in patients with disease progression compared with patients with no change or response (*P* = 0.027). The box plots depict the minimum and maximum values (whiskers), the upper and lower quartiles, and the median.

Finally, we sought to integrate the 3 sets of epigenomic data described above to build a robust cf-ChIP classifier for tRCC (Supplemental Table 1). Our tRCC classifier utilized 3 distinct cell line–informed signatures: H3K4me3 tRCC-up sites, H3K27ac tRCC-up sites, and TFE3 fusion–occupied binding sites (Supplemental Tables 2 and 3). Aggregating plasma H3K4me3 signal across H3K4me3 tRCC-up sites (*n* = 2,450) distinguished tRCC from ccRCC plasma samples (*n* = 27 and 11, respectively) with an AUC of 0.78. Aggregating plasma H3K27ac signal across H3K27ac tRCC-up sites (*n* = 11,443) and TFE3 fusion–occupied binding sites (*n* = 6,540) achieved AUCs of 0.8 and 0.84, respectively. To enhance the performance of the classifier, we combined the 3 scores for each sample (see Methods) to create a tRCC integrated epigenomic score (TIES) (Figure 3D). In this process, we ensured that signals at overlapping sites between H3K27ac tRCC-up sites and TFE3 fusion–occupied binding sites were not double counted (Supplemental Figure 2). This approach achieved an AUC of 0.86 for the discrimination of tRCC from ccRCC (Figure 4A). For discriminating tRCC from healthy plasma samples (*n* = 27 and 9, respectively), aggregated H3K4me3 signal at H3K4me3 tRCC-up sites, aggregated H3K27ac signal at H3K27ac tRCC-up sites, and TFE3 fusion–occupied binding sites achieved AUCs of 0.7, 0.89, and 0.87, respectively, with an integrated AUC of 0.92 (Figure 4A).

Leave-one-out cross-validation (LOO-CV) performed on tRCC and healthy plasma samples (see Methods) achieved a precision of 100%, a recall of 77.8%, and a specificity of 100%. The optimal actionable cutoff maximizing the difference between true positive and false positive rates, averaged across all LOO iterations, was 7.75. In addition, at the single-patient level, and among plasma samples with detectable ctDNA, 5 out of 6 patients with tRCC exhibited elevated (>7.75) TIESs. Interestingly, two tRCC plasma samples had markedly elevated TIESs but < 3% tumor fraction estimated by ichorCNA, possibly due to the paucity of copy number alterations in some tRCC tumors that may limit quantification of cfDNA quantity by tumor fraction alone (Supplemental Figure 5).

Given this observation, we next estimated the limit of detection of the TIES via in silico dilution. We combined the 10 tRCC samples with tumor fraction > 3% (as estimated by ichorCNA) with each of 9 healthy plasma samples at 10 dilution ratios (90 combinations for each of 10 dilution ratios ranging from 0.9 to 0.01 of tumor/healthy plasma by number of reads). Diluted samples were binned into intervals of 0.4% expected tumor fraction (ranging from <0.4% to >3.2%). In each bin, the TIES for the tumor dilutions was compared with the TIES for the 9 healthy plasma samples via Wilcoxon’s test. A significant difference between healthy samples and tRCC samples (*P* < 0.05) was observed down to a tumor fraction of 0.8%–1.2% (Figure 4B and Supplemental Figure 5). While previous studies have directly identified gene fusions in ctDNA using high-depth sequencing (49), the low coverage of our cfDNA low-pass whole-genome sequencing (cf-lpWGS) data (~0.1×) was insufficient to detect fusion-supporting reads; nonetheless, transcriptional evidence of the translocation could be detected via cf-ChIP with a tumor fraction in the 1% range. Finally, we also compared this approach to nucleosome depletion signatures at TFBSs in cfDNA using low-pass whole-genome sequencing (49). We used Griffin (50) to calculate the aggregated normalized coverage at the TFE3 fusion–occupied TFBS. No difference was observed between the median profile of ccRCC, tRCC, and that of individuals acting as healthy controls. However, the central coverage (defined as the bottom peak value) between subgroups showed a trend toward lower values for tRCC samples with a tumor fraction > 3% (Supplemental Figure 5).

Having established a tRCC-specific epigenetic signature that can be detected in plasma cfDNA, we also aimed to monitor tRCC disease burden via TIES measured by cf-ChIP in 3 patients with metastatic tRCC whose plasma samples were collected at multiple time points during treatment (Figure 4C and Supplemental Figure 6). In all 3 patients, we observed that variations in TIES were concordant with the clinical course of response and progression to systemic therapy. For instance, in the patient TRCCP4, we observed an increase of TIES at 3 time points (13, 26, and 52 months after diagnosis) corresponding to radiographic progression but a decrease at time points corresponding to disease control or response (18 months and 39 months after diagnosis) (Figure 4C). Interestingly, we also observed an increase in TIES within 3 months prior to the scan showing progression, although the ctDNA fraction remained undetectable at this time by cf-lpWGS. In patient TRCCP5 (initially misdiagnosed as ccRCC on pathology), we observed an increase in TIES at disease recurrence, followed by a subsequent decrease after a change in systemic therapy that resulted in radiographic disease control (Supplemental Figure 6). Similarly, in patient TRCCP3, we observed an initial decrease in TIES following curative nephrectomy, followed by an increase of the signal 16 months later, aligned with disease recurrence and metastasis to the liver (Supplemental Figure 6). We note that, across multiple patients, TIES was detectable and dynamic even when the tumor fraction was in the undetectable range (<3%) by a method that estimates ctDNA using copy number alterations (17).

To evaluate cf-ChIP for monitoring tRCC treatment response, we calculated changes in TIES and tumor fraction for each pair of consecutive plasma draws. When we compared these changes during intervals of disease progression, stability, or response, we found that TIES between consecutive draws increased at times of disease progression and decreased during response or disease stability (*P* = 0.027; Figure 4D). Importantly, changes in tumor fraction were far less pronounced (*P* = 0.63; Supplemental Figure 6). This suggests that our liquid biopsy epigenomic assay may be more effective at tracking disease evolution and response compared with other methods that rely solely on copy number alterations, likely due to the low frequency of such alterations in tRCC.

Finally, to evaluate the extensibility of our approach, we assessed its potential to detect other fusion-specific epigenomic signatures in plasma samples. We compared plasma samples from patients with prostate cancer with (*n* = 5) or without (*n* = 8) the *TMPRSS2-ERG* fusion, previously profiled with cf-ChIP (22). The *TMPRSS2-ERG* fusion, which places the *ETS* family transcription factor *ERG* under the control of the androgen-responsive gene TMPRSS2, is found in approximately 50% of prostate cancer cases and is associated with a distinctive transcriptional signature (51, 52). We observed that plasma from patients with the *TMPRSS2-ERG* fusion exhibited significantly higher H3K27ac signal at fusion-specific H3K27ac sites (*n* = 7,531) (53) compared with samples from patients with fusion-negative cancers (*P* = 0.006); this corresponded to an AUC of 0.95 for discriminating between samples with and without the *TMPRSS2-ERG* fusion (Supplemental Figure 7).

We also reasoned that cf-ChIP might be more broadly applicable in cancers with distinctive transcriptional profiles, such as those harboring driver fusions involving a transcription factor. To nominate additional cancer types that may be amenable to profiling via cf-ChIP, we first performed a pan-cancer survey of the fraction of genome altered (FGA), a metric of the proportion of the genome affected by copy number alteration (54). We observed substantial variation in FGA both between and within cancer lineages (Supplemental Figure 7); we note that tumors with low FGA may have insufficient CNAs to enable accurate estimation of tumor fraction in cfDNA (17). We then assessed how FGA tracked with fusion status across cancer types. When limiting to tumors harboring driver fusions involving transcription factors (55, 56) (analogous to the *TFE3* fusions in tRCC), we observed that fusion-positive cancers had significantly lower FGA than fusion-negative cancers (median 0.06 vs. 0.20, respectively, *P* < 2.2 × 10^–16^; Supplemental Figure 7), consistent with a prior pan-cancer fusion analysis, suggesting that >1% cancers may harbor a fusion oncogene as the sole driver (56). For example, FGA in *TFE3* fusion-positive RCCs was significantly lower than in other RCCs (median 0.08 vs. 0.15, respectively, *P* = 0.038). Similarly, FGA in SSX2 fusion-positive synovial sarcoma was significantly lower than in other sarcomas (median 0.08 vs. 0.34, respectively; *P* = 0.011) (Supplemental Figure 7). Together, these findings may suggest a potential applicability of cf-ChIP to an array of mutationally quiet cancers, particularly those harboring driver fusions involving a transcription factor.

## Discussion

Plasma epigenomic profiling offers a promising tool for accurate, sensitive, and noninvasive detection of tRCC. Liquid biopsy assays based on tumor genetic changes are challenging to adapt to tRCC, given that it harbors few recurrent genetic mutations and often displays few or no large-scale copy number alterations assessable via low-coverage genome-wide cfDNA sequencing approaches (9, 17, 18). Another challenge for tRCC detection in plasma is the wide variability of fusion partners and fusion breakpoints (18), which complicates the ability to apply a universal ultrasensitive targeted sequencing approach to this cancer. By contrast, plasma epigenomic profiling enables the inference of tumor-specific regulatory element activity from blood and detection of the TFE3 fusion-driven cistrome in plasma cfDNA regardless of fusion partner or specific breakpoint. Using in silico dilution of cf-ChIP reads, we show that tRCC ctDNA can be detected from circulating chromatin at tumor fractions of approximately 1%. To our knowledge, this is the first study to employ a noninvasive epigenomic profiling method for accurate detection and monitoring of tRCC in plasma, with potential implications for accurate diagnosis, prognostication, and therapy selection. Importantly, this approach can be broadly applied to other cancers that have distinctive epigenomic profiles driven by a transcription factor, but which lack sufficient genetic alterations to detect ctDNA with mutation-based methods.

Our findings have several clinical implications. First, underdiagnosis and misdiagnosis of tRCC presents a major challenge, stemming from its histological similarities with other RCC subtypes coupled with the absence of routine molecular testing in clinical practice. Proper diagnosis of tRCC is critical, given that it carries a worse prognosis than other RCC subtypes and that it may have higher metastatic potential and a lower response rate to systemic therapies typically used for ccRCC (9, 57–61). Second, accurate diagnosis of tRCC can help in optimal therapy selection for metastatic disease. While systemic therapies developed for ccRCC are often deployed to patients with tRCC, these therapies typically have lower response rates in tRCC owing to its distinct biology. For instance, belzutifan (HIF-2α inhibitor) has limited mechanistic rationale in tRCC, given that tRCC does not harbor alterations in *VHL* (62). Third, the ability to more accurately detect and diagnose tRCC would have implications for clinical trial enrollment — both to select patients for clinical trials specifically designed for tRCC and to prevent patients with tRCC from being inadvertently enrolled in trials designed for ccRCC, as has occurred with appreciable frequency in the past (9). Finally, we also demonstrate that clinical progression can be preceded by increases in tRCC epigenomic signals detected by cf-ChIP, even at a small systemic disease burden. This assay could therefore improve disease monitoring and inform early treatment switching. It may also be of use for risk stratification and counseling regarding adjuvant therapy. Adjuvant pembrolizumab following nephrectomy is approved for ccRCC but remains unproven and untested in other tumor subtypes like tRCC (63–65), typically being reserved for patients thought to be at highest risk of recurrence and who are most likely to benefit (64). Finally, the limitations of our study include the small sample size and the absence of a validation cohort, both attributable to the rarity of tRCC.

To date, plasma epigenomic profiling has primarily been used to discriminate tumors of distinct anatomical origin (22, 66, 67) and cancers with clear histological differences, for example, neuroendocrine transformation and lineage plasticity in prostate cancer or lung adenocarcinoma (25, 27, 29, 68) or sarcomatoid differentiation in RCC (28). Our study demonstrates that epigenomic profiling of plasma can also accurately distinguish between cancers with overlapping or even indistinguishable histologies, such ccRCC and tRCC. cf-ChIP-seq is distinctively suited to this purpose because it can detect the activation of regulatory elements by disease-driving transcription factors. Our method overcomes the challenges of detecting gene fusions from ctDNA by measuring the epigenomic effects of TFE3 activation, rather than rearrangement itself. This study has broad applicability to other cancers, particularly those driven by activation of a transcription factor and those that exhibit few copy number alterations and are thus underdetected using DNA alteration-based methods. Our study demonstrates the potential of next-generation liquid biopsy assays that identify transcriptional subtypes of cancer based on epigenomic signatures.

## Methods

### Sex as a biological variable

In our study, sex was not considered as a biological variable.

### Cell lines

The cell lines 786-O (ATCC, CRL-1932; RRID: CVCL_1051), 293T (ATCC, CRL-11268; RRID: CVCL_0063), A-498 (ATCC, HTB-44; RRID: CVCL_1056), 769-P (ATCC, CRL-1933; RRID: CVCL_1050), KMRC-1 (JCRB1010; RRID: CVCL_2983), Caki-1 (ATCC, HTB-46; RRID: CVCL_0234), UOK109 (RRID: CVCL_B123), and UOK146 (RRID: CVCL_B123) were obtained from W. Marston Linehan’s laboratory (National Cancer Institute). FU-UR-1 (RRID: CVCL_6997; Masako Ishiguro’s laboratory, Fukuoka University School of Medicine, Fukuoka, Japan) and s-TFE (RIKEN, RCB4699; RRID: CVCL_6997) were grown at 37°C in DMEM supplemented with 10% FBS, 100 U/mL penicillin, and 100 μg/mL Normocin (Thermo Fisher Scientific, NC9390718).

### Patient cohort

Plasma samples were collected from patients with tRCC (5 male and 5 female patients) and ccRCC (7 male and 5 female patients) diagnosed and treated at the Dana-Farber Cancer Institute (DFCI) between 2005 and 2024. All patients provided written informed consent. The collection and use of samples was conducted under an IRB-approved protocol at DFCI. Studies were conducted in accordance with recognized ethical guidelines. Plasma samples from healthy individuals without a history of diabetes, cancer, or major medical illnesses were reanalyzed from historical data generated using the same experimental protocol at DFCI, as previously reported (22).

### ChIP-seq sample processing

H3K27ac and TFE3 ChIP-seq data in tRCC cell lines that were previously generated in-house (44) are available in the Gene Expression Omnibus (GEO) under accession number GSE266530. H3K4me3 and H3K27ac ChIP-seq for ccRCC cell lines (Caki-1, A-498, RFX393, and 786-O) were reanalyzed from Gopi and Kidder (69) (GSE143653).

H3K4me3 ChIP-seq was performed on tRCC cell lines UOK146, s-TFE, and FU-UR-1 as previously described (39). Briefly, 3 × 10^6^ cells per reaction were collected, cross-linked with 1% formaldehyde, and quenched with 0.125 M glycine. After washing with ice-cold PBS, the pellet was resuspended in 130 μL SDS lysis buffer. Lysate was transferred to a Covaris E220 and sonicated to 200–500 bp. Samples were precipitated and centrifuged, and 100 μL of each sample was diluted 10 times in ChIP dilution buffer and used downstream. Ten microliters of Dynabeads protein G and 10 μL of protein A were washed and complexed with 10 μL of H3K4me3 antibody (rabbit mAb 9751, Cell Signaling; RRID: AB_2616028) and incubated with the lysate at 4°C overnight. Cells were pelleted and washed in 1 mL of each of the cold buffers in the order listed below on a rotating platform at 4°C followed by brief centrifugation and removal of the supernatant fraction on a magnetic rack. The cold buffers were as follows: (a) low-salt Immune Complex Wash Buffer, 1 wash for 5 min; (b) High-Salt Immune Complex Wash, 1 wash for 5 min; (c) LiCl Immune Complex Wash Buffer, 1 wash for 5 min; and (d) TE, 2 washes for 5 min. ChIP DNA was reverse cross-linked and purified for DNA library construction using the KAPA HyperPrep Kit (KAPA Biosystems, KR0961).

MeDIP-seq was performed on cell lines (UOK146, s-TFE, FU-UR-1, Caki-1, A-498, 786-O, 786-P, and KMRC-1) as previously described (23).

For cfDNA samples, processing for H3K4me3/H3K27ac cf-ChIP and cf-MeDIP-seq on plasma samples was also performed as previously reported (22, 23). Quality metrics for sequencing are provided in Supplemental Table 4.

### Analysis of cell line ChIP-seq and MeDIP-seq data

#### Peak calling and identification of tRCC-up regions.

For cell line MeDIP-seq and H3K4me3/H3K27ac ChIP-seq data, peak calling was performed using the ChiLin computational pipeline that automates the quality control and data analyses of ChIP-seq (70), with the ChiLin command simple and the parameters –p narrow, -r histone, and hg38 as the reference genome. For differential peak analysis between tRCC and ccRCC cell lines, a consensus peak file was generated using DiffBind (version 3.10.1; RRID: SCR_012918) (38), and integrated DESeq2-based differential peak calling was performed with the DiffBind (38) command dba.analyze. Differentially marked sites were deemed significant if the FDR *q* value was < 0.01. We then focused on sites exhibiting a log_2_FC in either direction greater than 1 for H3K27ac and greater than 2 for H3K4me3.

#### Identification of fusion-occupied TFE3 TFBSs.

For determination of fusion-occupied TFE3 TFBSs, we first downloaded known TFE3 (WT) TFBSs from the GTRD (Homo_sapiens_meta_clusters.zip, http://gtrd.biouml.org:8888/downloads/current/intervals/chip-seq/) (71). This database contains a compilation of ChIP-seq data from various sources with accurate TFBS positions defined by a combination of experiments from various cell lines and 4 calling algorithms (MACS2, SISSR, GEM, and PICS).

To select TFBSs that remained active across TFE3 fusions regardless of fusion partner, we determined a set of high-confidence common peaks between WT TFE3 and various TFE3 fusions. For this, we overlapped the 24,050 peaks available in the GTRD originating from 2 non-RCC cell lines (Lovo and HepG2) with 29,785 peaks identified by the union of TFE3 ChIP-seq in tRCC cell lines (FU-UR-1, UOK109, and s-TFE) (44) to determine a set of 6,540 fusion-occupied TFBSs. Overlap of peaks was performed using BEDTools v2.27.1 (RRID: SCR_006646) (72). Peaks were considered overlapping if they shared 1 or more base pairs.

All region sets were then lifted over from hg38 to hg19 using the UCSC tool Lift Genome Annotations (https://genome.ucsc.edu/cgi-bin/hgLiftOver) for comparison with plasma samples.

Profile plots of H3K27ac signal at TFE3 TFBSs in RCC cell lines were generated using deepTools version 3.5.5 (73) (RRID: SCR_016366).

#### Identification of DMRs from TCGA data.

Raw IDAT files were processed using the SeSAMe package to obtain normalized methylation β values. Probes were filtered to exclude those flagged by general quality masks, non-CpG sites, and sex chromosome–associated probes. Differentially methylated probes were identified using the limma package, which applies linear modeling with empirical Bayes moderation of standard errors to improve variance estimation and statistical power. For each comparison, probes with adjusted *P* values < 0.05 and an absolute methylation difference ≥ 0.3 were selected as significantly hyper- or hypomethylated. These CpG sites were mapped to genomic coordinates using the Illumina 450K hg19 annotation. To define robust methylated regions, we extended each CpG site by ±500 bp and merged overlapping intervals using the IRanges package. The resulting nonoverlapping regions were exported as BED files for downstream cf-MeDIP-seq signal quantification.

### Plasma cf-ChIP

H3K27ac and H3K4me3 cf-ChIP was performed serially on plasma samples using previously published methods (22). The following antibodies were used: H3K27ac (Abcam ab4729) and H3K4me3 (Thermo Fisher Scientific, PA5-27029).

### Processing and analysis of cfDNA

#### Tumor fraction calculation.

cf-lpWGS was performed on plasma samples using previously published methods (22). The ichorCNA R package (RRID: SCR_024768) was used to infer copy number profiles and cfDNA tumor content from read abundance across bins spanning the genome using default parameters (17). For plasma samples without cf-lpWGS data, we used signal at previously described circulating regulatory elements to estimate the cfDNA tumor fraction (22).

#### Analysis of cf-ChIP-seq and cf-MeDIP-seq data.

H3K4me3/H3K27ac cf-ChIP-seq and cf-MeDIP-seq reads were aligned to the hg19 human genome build using Burrows-Wheeler Aligner version 0.7.1740 (74) (RRID: SCR_010910). Nonuniquely mapping and redundant reads were discarded. MACS version 2.1.1 (RRID: SCR_013291) (75) was used for ChIP-seq peak calling with a *q* value (FDR) threshold of 0.01. Fragment locations were converted to BED files using BEDTools (72) (version 2.29.2) using the bamtobed command with the -bedpe flag set. For analyses involving overlap with genomic regions such a differentially marked regions or TFE3 binding sites, fragments were imported as GRanges objects and collapsed to 1 bp at the center of the fragment location to ensure that a fragment could map to only 1 site.

#### Quantification of peaks and TFBSs in plasma.

We inferred transcriptional activity at sites of interest based on H3K27ac and H3K4me3, as described previously (22). Briefly, peaks were resized to a 3-kb interval centered on the original peak, then binned into 40 bp windows. Fragment counts were aggregated across each 40 bp window for all peaks to obtain aggregate profiles for each sample. To account for variation in background signal across samples, we performed a shoulder normalization step, as previously described (22). We also normalized signal in each bin to the aggregated signal at the common 10,000 DNAse hypersensitivity sites that are expected to be active across most tissue types and defined across the largest number of samples by Meuleman et al. (76). Similar analysis was used to assess cfDNA methylation patterns at tRCC-DMRs while normalizing signal to the total number of fragments in each sample.

#### Detection of tRCC epigenomic signature in plasma.

Our feature set included the differentially marked regions (H3K4me3 and H3K27ac tRCC-up peaks) and TFE3 fusion–occupied binding sites, described above. We measured H3K27ac/H3K4me3 cf-ChIP-seq signal at these sites as described above. Subsequently, for each sample, we summed the 3 signals and applied a log transformation to derive a single score, referred to as the TIES. In this process, we discarded 853 peaks, which were shared between H3K27ac tRCC-up peaks and the TFE3 fusion–occupied binding sites, from the TFE3 fusion–occupied binding sites to avoid double counting of the signal. The classifier performance was assessed by measuring the area under the receiver operating characteristic (ROC) curve (27), using ROCR R package. In brief, the ROC curve was constructed by plotting the true positive rate (sensitivity) against the false positive rate (1 – specificity) for various decision thresholds. The AUC was then calculated to summarize the overall performance of the classifier.

### LOO-CV

LOO-CV was performed on all available tRCC and healthy plasma samples with a TIES (*N* = 36; 27 tRCC, 9 healthy). For each iteration, 1 plasma sample was left out, and Youden’s index (True positive rate – False positive rate) was calculated on the remaining 35 samples to identify the TIES threshold that maximized the index. This optimal threshold was then applied to the TIES of the left-out sample to assign a prediction: samples with scores above the threshold were classified as tRCC, and those below were classified as healthy. After all 36 samples were individually evaluated, the predicted labels were compared with the true labels, and performance metrics, including recall (sensitivity), precision, and specificity, were calculated.

### Processing of samples for CTC isolation

#### CTC processing.

CTCs were isolated from freshly collected whole-blood samples as previously described (77). To ensure high recovery of intact CTCs with quality RNA, blood was processed within 4 hours of collection. Leukocytes were depleted using the microfluidic CTC-iChip system (TellBioDX). Whole blood was treated with biotinylated antibodies targeting CD45 clone HI30 (Thermo Fisher Scientific, MHCD4505; RRID: AB_10372216), CD66b clone 80H3 (Standard BioTools, 3162023B; RRID: AB_3661862), and CD16 (BD Biosciences, clone 3G8), followed by incubation with Dynabeads MyOne Streptavidin T1 (Invitrogen) for magnetic labeling and depletion of white blood cells. After enrichment using the CTC-iChip, the CTC product was kept on ice, centrifuged at 1,000*g*, and flash-frozen in liquid nitrogen with RNAlater (Ambion) for downstream expression analysis.

#### RNA extraction, whole-transcriptome amplification, and ddPCR.

RNA was extracted from CTC samples using the RNeasy Plus Micro Kit (Qiagen) following manufacturer’s protocol. Whole-transcriptome amplification was carried out on RNA samples using SMARTer Pico PCR cDNA, following the manufacturer’s protocol with 18–21 amplification cycles.

#### Transcript target selection and primer design.

Transcripts of interest selective for tRCC or ccRCC were selected based on the analysis of RNA-seq data as described in the manuscript. RNA-seq data for ccRCC cell lines were downloaded from The Cancer Dependency Map Portal (https://depmap.org/portal/download/). RNA-seq data for tRCC cell lines were previously generated in-house (44) and are under accession number GSE266517. Data for white blood cells were obtained from GTEX V8 (https://gtexportal.org/home/downloads/adult-gtex/bulk_tissue_expression). Transcript targets were selected based on high expression in tRCC or ccRCC (Supplemental Figure 2) and absence of expression in white blood cells defined by <0.5 transcripts per million in GTEX samples. Primers used for ddPCR for specific target genes were obtained from Bio-Rad: *EEF1G*, 10031252-dHsaCPE5191671; *TRIM63*, 10031255-dHsaCPE5049837; *GPR143*, 10031252-dHsaCPE5058198; and *C1QL1*, 10031252-dHsaCPE5052250.

#### ddPCR.

cDNA and primer/probe mixes were combined with ddPCR Supermix for Probes (Bio-Rad) in a 96-well plate and loaded into Bio-Rad’s automated droplet generator. Droplets were then subjected to thermal cycling, and droplets containing the target transcripts were detected via fluorescence using the QX200 Droplet Reader System (Bio-Rad).

### cf-ChIP analysis of TMPRSS2-ERG fusion-positive prostate cancer samples

Previously profiled in-house plasma samples from patients with metastatic prostate adenocarcinoma (available in GEO under accession number GSE243474) (22) that had matched panel sequencing data available from the same year as plasma sample were included in the analysis. H3K27ac signal was aggregated at sites differentially marked in fusion-positive compared with fusion-negative prostate cancer identified previously (78), as described in *Quantification of peaks and TFBSs in plasma*. The classifier performance to distinguish fusion-positive from fusion-negative prostate cancer was assessed by measuring the AUC, as previously described (27).

### In silico dilution

cf-ChIP-seq reads from tRCC plasma samples with a tumor fraction > 3% (*n* = 10) and healthy plasma samples with available cf-lpWGS data (*n* = 9) were randomly selected. The samples were mixed, creating all possible pairwise combinations (*n* = 90) at each dilution level, ranging from 0.9 to 0.01, using Samtools. For each dilution of a tRCC and healthy sample pair, the sample with the fewer reads, S1 (compared with its counterpart S2), was used to set the read number reference, N, to perform the dilution. At a given dilution level (DL), NxDL reads were randomly sampled from S1 and mixed with Nx(1-DL) reads from S2. The median number of reads combined was 9,331,731 (IQR: 5,228,110–12,071,840).

### TCGA analysis

Fraction genome altered annotations were obtained from the TCGA database for 10,767 tumor samples from 25 cancer types (79) via cBioPortal (80). Driver fusions were taken from a previously published pan-cancer consensus set (56) and limited to those involving transcription factors (55), in analogy to *TFE3* fusions in tRCC. In the pan-cancer analysis, the FGA was compared between fusion-negative and fusion-positive cancers across all cancer types. In the analysis of *TFE3* fusion in RCC and *SSX2* fusion in synovial sarcoma, we compared fusion-positive RCC and synovial sarcoma with other RCCs and sarcomas without fusions, respectively.

### Statistics

Statistical analysis was conducted using Wilcoxon’s rank sum test, with a significance level set at 0.05. All analyses were performed using R (version 4.4) on RStudio (version 2024.04.0). A *P* value of less than 0.05 was considered statistically significant.

### Study approval

All patients provided written informed consent. The collection and use of samples were conducted under an IRB-approved protocol at DFCI. Studies were conducted in accordance with recognized ethical guidelines.

### Data availability

Raw data (FASTQ files) and processed data (BED and BigWig files) are available through GEO under accession numbers GSE280708, GSE266530, and GSE243474. The dataset used in this manuscript is available in the Supporting Data Values file. Algorithms used for data analysis are all publicly available from the indicated references in Methods.

## Author contributions

SG, KS, TKC, MLF, SCB, and SRV conceived the study. SG and KS led data analysis with assistance from JL, AS, BJF, P Konda, MA, and MP under the joint supervision of SCB and SRV. JC, NP, HS, MPD, KL, AM, and RN performed experiments with input and/or supervision from KK, JHS, BDC, MLF, and SRV. JO, JH, DF, CHC, WDF, JEB, RT, P Khanna, and TKC assisted in procuring clinical samples. ZZ and REHC assisted with processing of cf-ChIP-seq data. SG, KS, SCB, and SRV wrote the first draft of the manuscript, and all authors participated in revision of the manuscript. Authorship order among co–first authors (SG and KS) was determined based on the relative contribution to conceptualization, data analysis, and manuscript writing, with both authors contributing equally overall. Authorship order among co–corresponding authors was determined based on the relative contribution to conceptualization, supervision of trainees, procurement of funding, and supervision of this study, with all co–corresponding authors contributing equally overall.

## Funding support

This work is the result of NIH funding, in whole or in part, and is subject to the NIH Public Access Policy. Through acceptance of this federal funding, the NIH has been given a right to make the work publicly available in PubMed Central.

- Doris Duke Charitable Foundation (Clinician Scientist Development Award, grant 2020101) (to SRV).
- Department of Defense Kidney Cancer Research Program (DoD KCRP) (W81XWH-19-1-0815 and W81XWH-22-1-016) (to SRV).
- Rally Foundation Independent Investigator Award (to SRV).
- National Foundation for Cancer Research (to SRV).
- V Foundation (V2022-018) (to SRV).
- National Cancer Institute (R01CA286652 and R01CA279044) (to SRV).
- DF/HCC Kidney SPORE DRP (2P50CA101942-16) (to SRV).
- ARC (Association pour la Recherche sur le Cancer) (to SG).
- La Ligue Contre le Cancer (to SG).
- Institut Servier (to SG).
- Philippe Foundation (to SG).
- Arthur Sachs grant (to SG).
- DoD KCRP Postdoctoral and Clinical Fellowship (HT94252310066) (to PK).
- DoD awards W81XWH-21-1-0299 to SCB and W81XWH-21-1-0234, W81XWH-21-1-0339, and W81XWH-19-1-0554 to MLF.
- NIH awards U01CA296432 to SCB and R01CA251555, R01CA227237, R01CA262577, and R01CA259058 to MLF.
- The Damon-Runyon Cancer Research Foundation (to SCB).
- The Kure It Cancer Research Foundation (to SCB).
- The Fund for Innovation in Cancer Informatics (to SCB).
- National Cancer Institute award U01 CA296432 (to SCB).
- Debbie and Bob First (to SCB).
- The Claudia Adams Barr Program for Innovative Cancer Research (to MLF).
- The H.L. Snyder Medical Research Foundation (to MLF).
- The Cutler Family Fund for Prevention and Early Detection (to MLF).
- The Donahue Family Fund (to MLF).
- Movember PCF Challenge Award (to MLF).
- Dana-Farber/Harvard Cancer Center Kidney SPORE (2P50CA101942-16) and Program 5P30CA006516-56 (to TKC).
- The Kohlberg Chair at Harvard Medical School (to TKC).
- The Trust Family (to TKC).
- Michael Brigham (to TKC).
- Pan Mass Challenge (to TKC).
- Hinda and Arthur Marcus Fund (to TKC).
- Roger and Kathy Marino Fund for Research Acceleration (to TKC).
- Frank Shaughnessy Kidney Cancer Research Fund (to TKC).
- Loker Pinard Funds for Kidney Cancer Research at DFCI (to TKC).

## Supplementary Material

Supplemental data

Supplemental table 1

Supplemental table 2

Supplemental table 3

Supplemental table 4

Supporting data values

## Version 1 02/02/2026

Electronic publication
