# Supplementary material for: Cell-free DNA epigenomic profiling enables noninvasive detection and monitoring of translocation renal cell carcinoma
Source: J Clin Invest. 2026 Feb 2;136(3):e195725. doi: 10.1172/JCI195725 (PMC12867156; doi:10.1172/JCI195725)
Supplement: Supplemental data [file jci-136-195725-s349.pdf]

SUPPLEMENTARY FIGURES

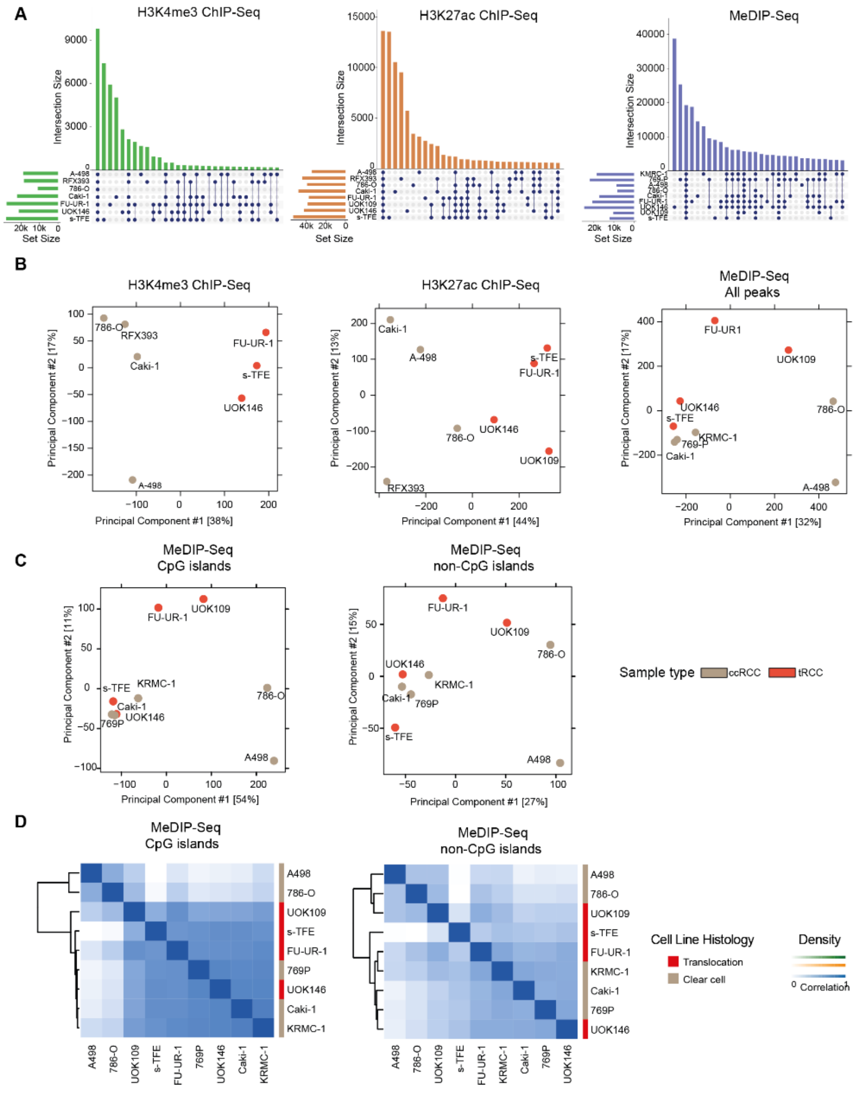

Figure S1

Supplemental Figure 1. Epigenomic analysis of tRCC and ccRCC cell lines to derive cell-line informed tRCC signature

(A) Upset plots showing the intersection of H3K4me3, H3K27ac, and MeDIP peaks across RCC cell lines analyzed in this study. tRCC cell lines: FU-UR-1, UOK146, UOK109, s-TFE; ccRCC cell lines: A-498, RFX393, 786-O, Caki-1, KMRC-1, 786-P).

(B) Principal-component analysis (PCA) plots of H3K4me3 ChIP-seq, H3K27ac ChIP-seq and MeDIP-seq peaks for RCC cell lines profiled in this study.

(C) Principal component analysis (PCA) and **(D)** unsupervised hierarchical clustering of MeDIP-seq peaks from RCC cell lines profiled in this study, restricted to CpG-islands (left) and non-CpG islands (right). tRCC and ccRCC samples are colored in red and grey, respectively.

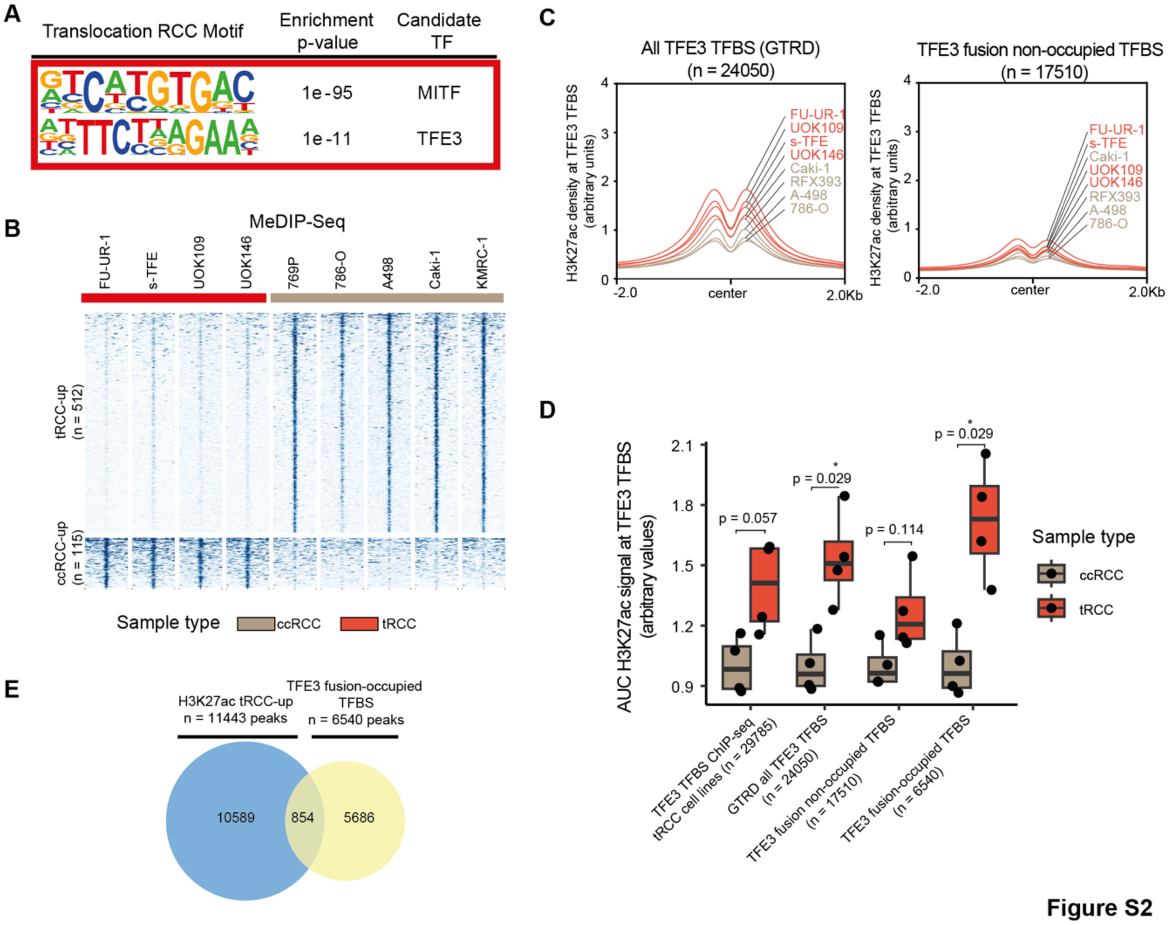

Figure S2

Supplemental Figure 2. Characterization of the tRCC-specific sites

(A) Motif analysis showing significant enrichment of MITF and TFE3 binding sites amongst 11,443 H3K27ac tRCC-up peaks.

(B) Heatmaps of normalized MeDIP tag densities at differential MeDIP-seq peak regions between tRCC and ccRCC cell lines (over a window ±2 kb from peak center).

(C) Aggregated H3K27ac signal density at all 24,050 TFE3 TFBS from GTRD (left) or 17,510 fusion non-occupied TFE3 TFBS in RCC cell lines (right).

(D) Boxplots of averaged H3K27ac signal at TFE3 TFBS determined respectively from TFE3 ChIP-seq in 3 tRCC cell lines (UOK109, s-TFE, FU-UR-1, N=29,785)), all TFE3 TFBS from GTRD database (N=24,050), TFE3 fusion non-occupied TFBS (N=17,510) or TFE3 fusion-occupied TFBS (N=6,540). P-values were determined by Wilcoxon test.

(E) Venn diagram showing overlap between H3K27ac tRCC-up peaks (determined by differential analysis of H3K27ac signal in tRCC and ccRCC cell lines) and TFE3 fusion occupied TFBS.

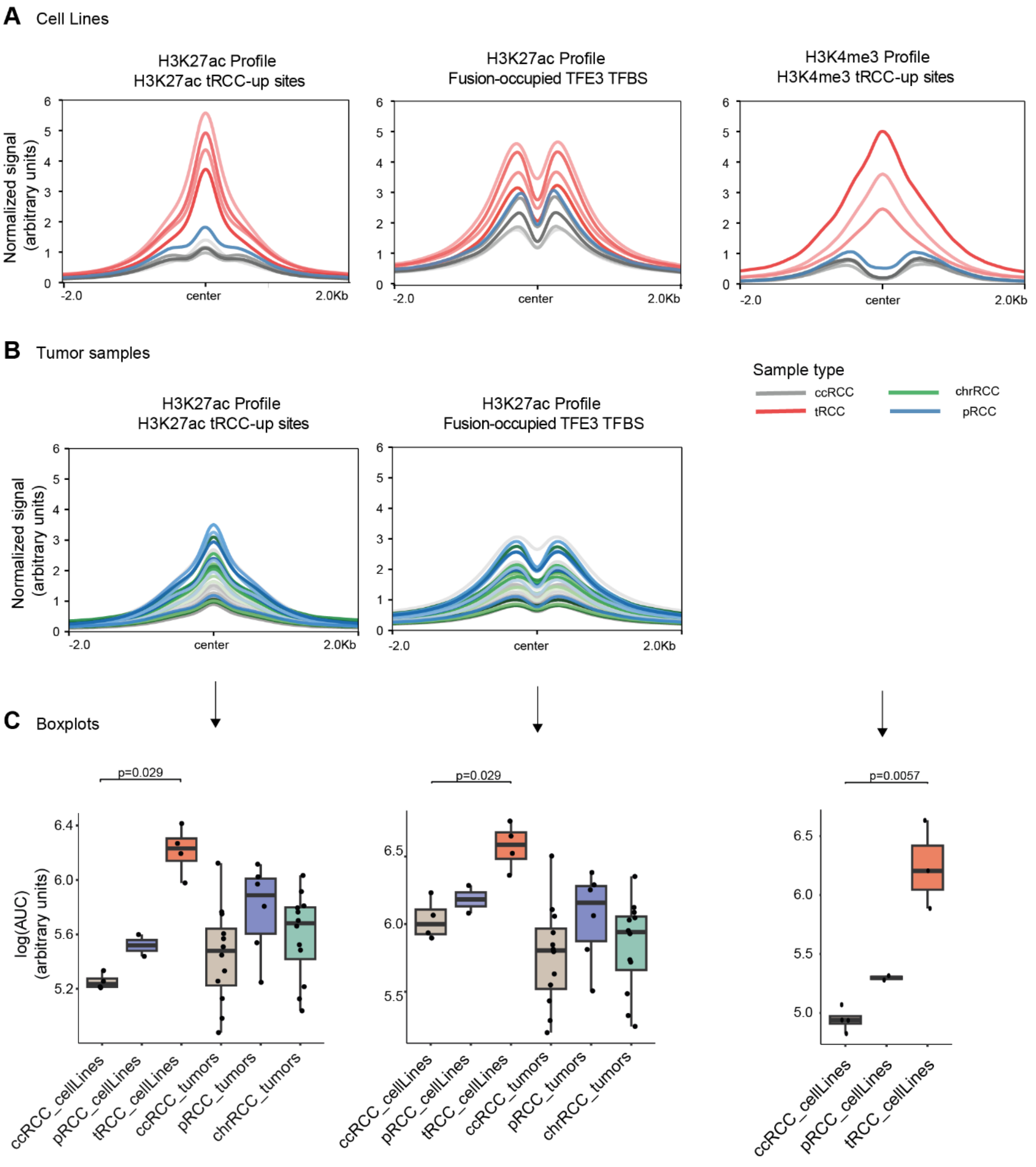

Figure S3

Supplemental Figure 3. Evaluation of the specificity of identified regions to tRCC vs. other RCC subtypes

Profile plots of normalized coverage plot at the H3K27ac-tRCC-up, fusion-occupied TFE3 TFBS, and H3K4me3-tRCC-up sites in RCC cell lines **(A)** and at the H3K27ac-tRCC-up, fusion-occupied TFE3 TFBS in RCC tumor samples of the indicated histologies (no tRCC tumors were available for analysis) **(B)**.

(C) Logarithm of the area under curve from the profile plots in A-B (Wilcoxon test). P-values were not calculated for tRCC vs pRCC due to the availability of only 2 pRCC cell lines.

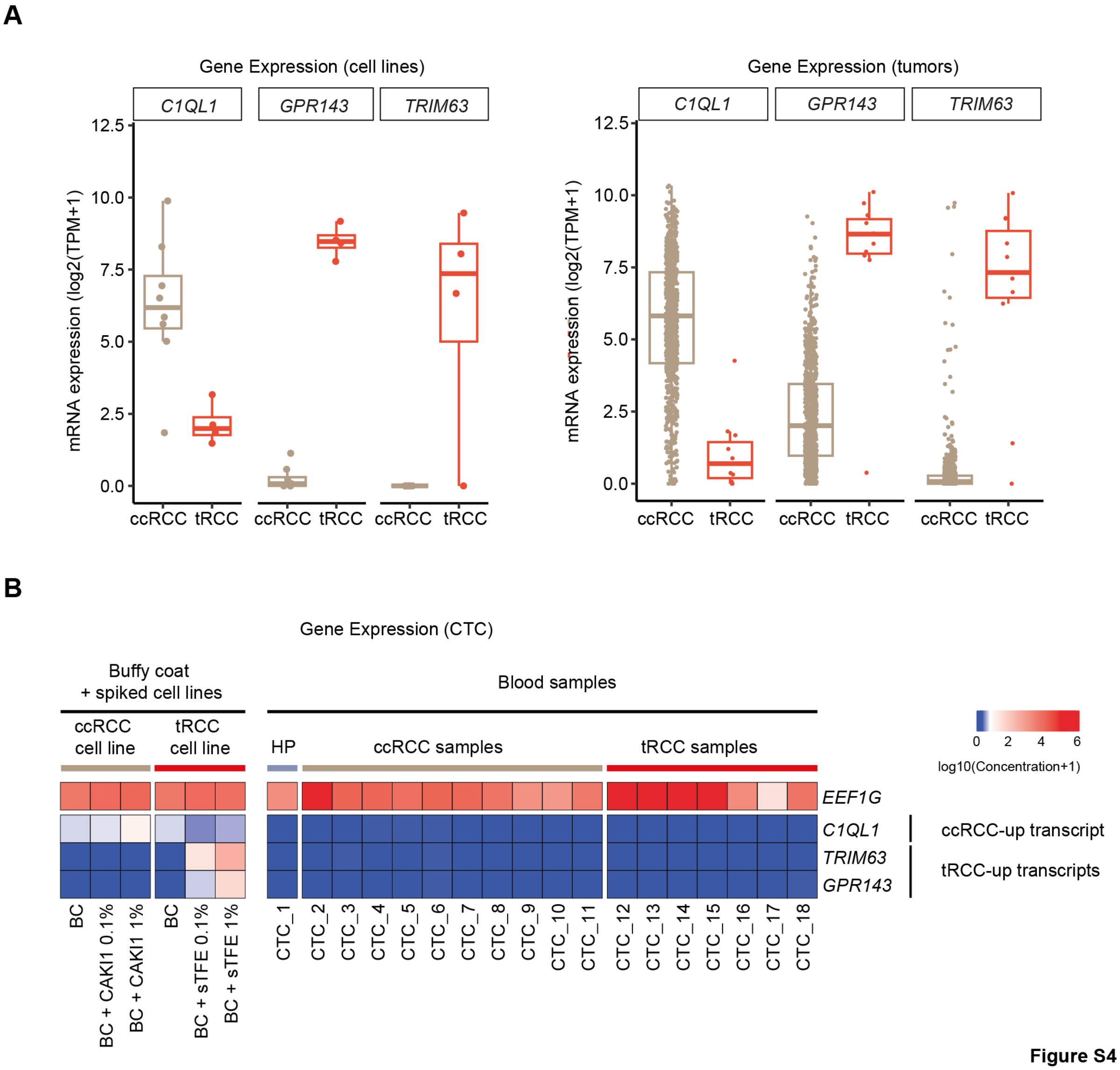

Figure S4

Supplemental Figure 4. Interrogation of tRCC-specific transcripts in cell lines, tumors, and CTCs

(A) Expression of tRCC-selective (*GPR143*, *TRIM63*), and ccRCC-selective (*C1Q1L*) genes in RNA-seq data of RCC cell lines from Depmap (tRCC: UOK109, UOK146, s-TFE, FU-UR-1; ccRCC: A-498, ACHN, Caki-1, Caki-2, KMRC-1, KMRC-2, KMRC-20, s786-O) (1) or ccRCC/tRCC tumors from a published study (2).

(B) Expression of target genes (constitutive: *EEF1G*; ccRCC-selective: *C1QL1*; tRCC-selective: *TRIM63, GPR143*) as detected by ddPCR in CTC isolates from the indicated patients or in healthy blood spiked with either 0.1% or 1% (by RNA quantity) of RNA derived from ccRCC cell line (Caki-1) or tRCC cell line (s-TFE).

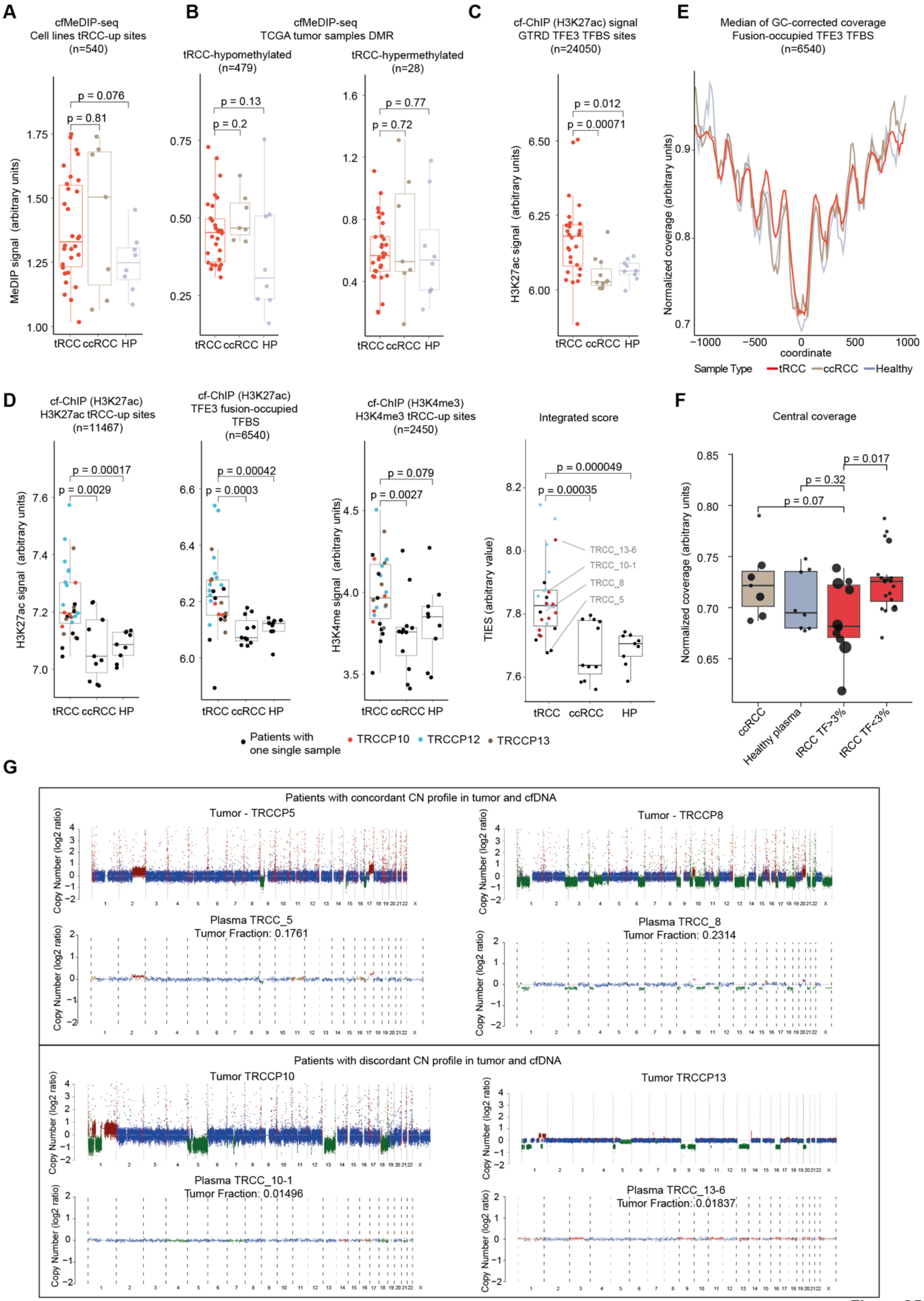
A
cfMeDIP-seq
Cell lines tRCC-up sites
(n=540)
MeDIP signal (arbitrary units)
p = 0.076
p = 0.81
tRCC ccRCC HP
B
cfMeDIP-seq
TCGA tumor samples DMR
tRCC-hypomethylated
(n=479)
p = 0.13
p = 0.2
tRCC-hypermethylated
(n=28)
p = 0.77
p = 0.72
C
cf-ChIP (H3K27ac) signal
GTRD TFE3 TFBS sites
(n=24050)
H3K27ac signal (arbitrary units)
p = 0.012
p = 0.00071
E
Median of GC-corrected coverage
Fusion-occupied TFE3 TFBS
(n=6540)
Normalized coverage (arbitrary units)
coordinate
Sample Type tRCC ccRCC Healthy
D
cf-ChIP (H3K27ac)
H3K27ac tRCC-up sites
(n=11467)
p = 0.00017
p = 0.0029
cf-ChIP (H3K27ac)
TFE3 fusion-occupied
TFBS
(n=6540)
p = 0.00042
p = 0.0003
cf-ChIP (H3K4me3)
H3K4me3 tRCC-up sites
(n=2450)
H3K4me signal (arbitrary units)
p = 0.079
p = 0.0027
Integrated score
TIES (arbitrary value)
p = 0.000049
p = 0.00035
TRCC_13-6
TRCC_10-1
TRCC_8
TRCC_5
Patients with one single sample
TRCCP10
TRCCP12
TRCCP13
F
Central coverage
Normalized coverage (arbitrary units)
p = 0.017
p = 0.32
p = 0.07
ccRCC
Healthy plasma
tRCC TF>3%
tRCC TF<3%
G
Patients with concordant CN profile in tumor and cfDNA
Tumor - TRCCP5
Tumor - TRCCP8
Plasma TRCC_5
Tumor Fraction: 0.1761
Plasma TRCC_8
Tumor Fraction: 0.2314
Copy Number (log2 ratio)
Patients with discordant CN profile in tumor and cfDNA
Tumor TRCCP10
Tumor TRCCP13
Plasma TRCC_10-1
Tumor Fraction: 0.01496
Plasma TRCC_13-6
Tumor Fraction: 0.01837

Figure S5

Supplemental Figure 5. Validation of cf-ChIP in to detect tRCC in plasma

(A) Aggregated MeDIP signal at cell-line informed tRCC-up MeDIP sites in tRCC, ccRCC, and healthy plasma samples. Statistical differences were determined by Wilcoxon test.

(B) Aggregated MeDIP signal at ccRCC vs tRCC differential methylated regions from TCGA, in tRCC, ccRCC, and healthy plasma samples. Statistical differences were determined by Wilcoxon test.

(C) Aggregated H3K27ac signal at all TFE3 TFBS peaks from GTRD (n=24,050) in tRCC, ccRCC, and healthy plasma samples. Statistical differences were determined by Wilcoxon test.

(D) Aggregated H3K4me3 and H3K27ac cf-ChIP signals at cell-line informed tRCC-up sites (left and middle-left), TFE3 fusion-occupied TFBS (middle-right) and integrated score (TIES, right) across all samples in this study, colored by patient. Samples from patients whose tumors were profiled via WGS (shown in D) are labelled (grey) in the rightmost panel. Statistical differences were determined by Wilcoxon test.

(E) Median GC-corrected agglomerated coverage from cf-lpWGS at fusion-occupied TFE3 TFBS (n = 6,540), computed using the Griffin pipeline.

(F) GC-corrected central coverage at fusion-occupied TFE3 TFBS (n = 6,540) for each plasma sample. tRCC samples are stratified by tumor fraction. The size of each dot is proportional to the tumor fraction. Comparisons were performed using the Wilcoxon rank-sum test.

(G) Comparison of genome-wide copy number profiles determined by WGS of plasma cfDNA (ichorCNA (3)) or via WGS of tumors (TITAN (4)), in four patients with matching plasma and samples. Tumor samples were profiled as part of a previously published study (5).

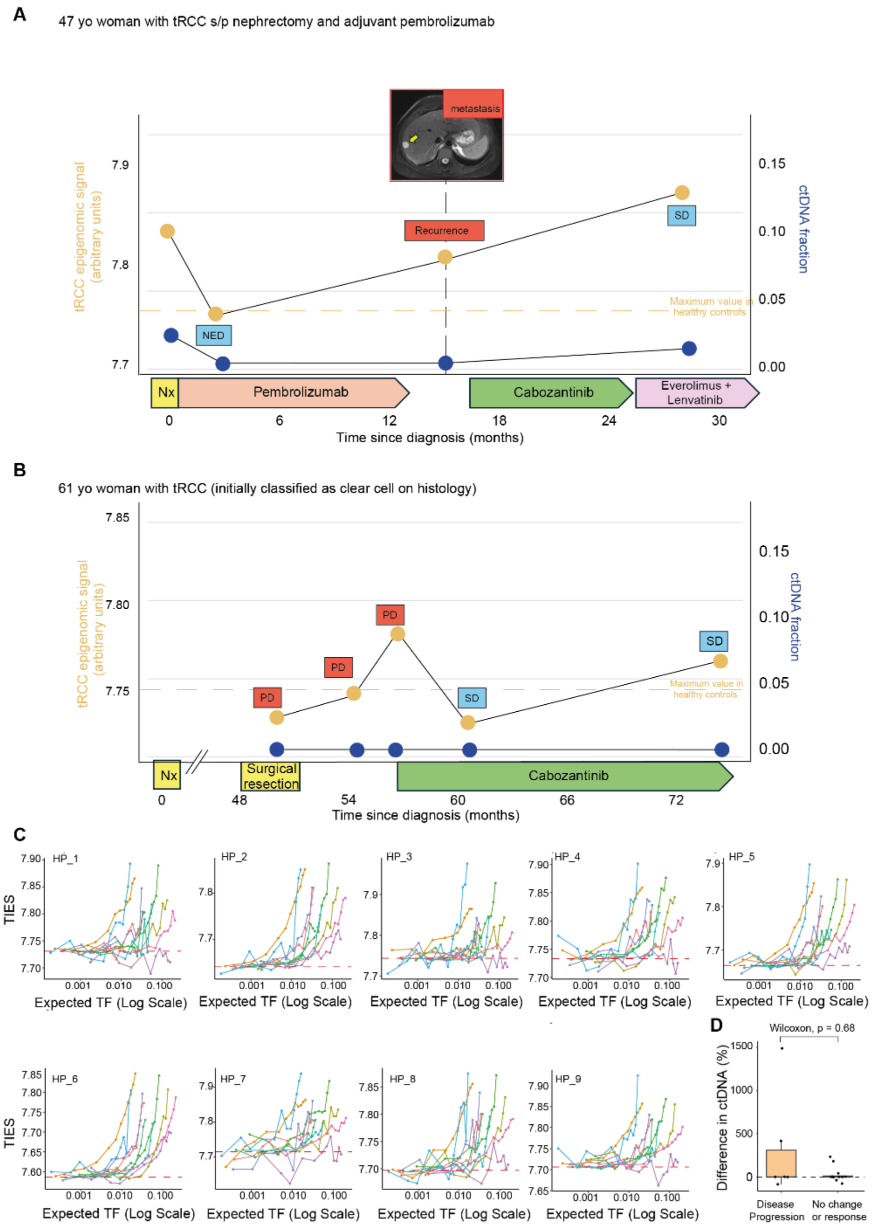

Figure S6

Supplemental Figure 6. Monitoring of tRCC using cf-ChIP

(A-B) Longitudinal tracking of the tRCC integrated epigenomic score (TIES, orange) and ctDNA fraction (blue) in two patients with tRCC. Pertinent radiographic and clinical milestones are indicated in the graphs. SD: stable disease; PR: partial response; PD: progressive disease; NED: no evidence of disease; Nx: nephrectomy.

(C) In silico dilution of tRCC cf-ChIP-seq reads from 10 tRCC plasma samples with detectable TF and the 9 healthy plasma samples. Each color represents a tRCC sample. TIES was calculated for every pair-wise combination of tRCC and healthy samples at the indicated dilutions. Red dashed line is the TIES of the one healthy control used for dilution. Each tRCC sample is plotted with a distinct color.

(D) Percentage change in CNA-based cell-free DNA tumor fraction (TF) between consecutive plasma draws grouped by radiographic response during that same interval. Statistical differences were determined by Wilcoxon test.

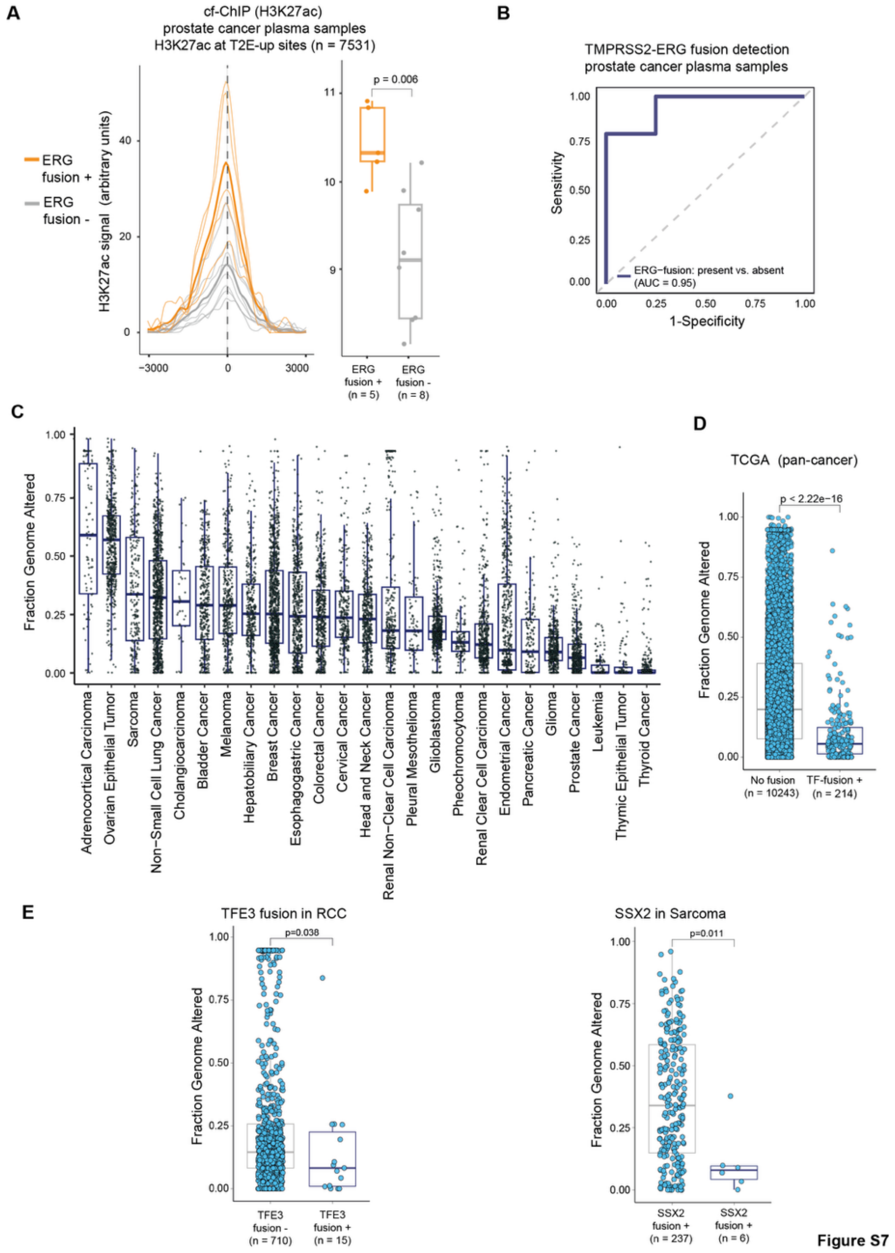

Supplemental Figure 7. Generalizability of the approach in fusion-driven, mutationally quiet cancers

(A) Aggregated cf-ChIP H3K27ac signal compared between *TMPRSS2-ERG* fusion-positive (n=5) and fusion-negative (n=8) prostate cancer plasma samples, at fusion-specific peaks. Statistical differences were determined by Wilcoxon test.

(B) Classifier assessing cf-ChIP H3K27ac signal at *TMPRSS2-ERG* fusion specific peaks in distinguishing fusion-positive and fusion-negative prostate cancer plasma samples.

(C) Fraction of genome altered (FGA) by cancer type across the TCGA.

(D) Distribution of FGA in cancers harboring a fusion involving a transcription factor versus fusion-negative cancers, across the TCGA.

(E) Distribution of FGA in *TFE3* fusion-positive RCC tumor samples vs. other RCCs (left) and SSX2 fusion-positive synovial sarcoma tumor samples vs. other sarcomas (right).

SUPPLEMENTARY TABLES

Table S1. Annotation of clinical cohort profiled in this study

Table S2. List of H3K27ac and H3K4me3 sites enriched in tRCC (“tRCC-Up”) or ccRCC (“ccRCC-Up”).

Table S3. List of consensus fusion occupied TFE3 binding sites used for integrated epigenomic score

Table S4. QC metrics of cf-Medip-seq, H3K4me3/H3K27ac cf-ChIP-seq

SUPPLEMENTARY REFERENCES

1. Tsherniak A, et al. Defining a Cancer Dependency Map. *Cell*. 2017;170(3):564.

2. Motzer RJ, et al. Molecular Subsets in Renal Cancer Determine Outcome to Checkpoint and Angiogenesis Blockade. *Cancer Cell*. 2020;38(6):803-817.e4.

3. Adalsteinsson VA, et al. Scalable whole-exome sequencing of cell-free DNA reveals high concordance with metastatic tumors. *Nat Commun*. 2017;8(1):1324.

4. Ha G, et al. TITAN: inference of copy number architectures in clonal cell populations from tumor whole-genome sequence data. *Genome Res*. 2014;24(11):1881–1893.

5. Achom M, et al. A genetic basis for sex differences in Xp11 translocation renal cell carcinoma. *Cell*. 2024;187(20):5735-5752.e25.
